# Effects of Nocturnal UV-B Irradiation on Growth, Flowering, and Phytochemical Concentration in Leaves of Greenhouse-Grown Red Perilla

**DOI:** 10.3390/plants10061252

**Published:** 2021-06-20

**Authors:** Hideo Yoshida, Tatsuru Nishikawa, Shoko Hikosaka, Eiji Goto

**Affiliations:** 1 Graduate School of Horticulture, Chiba University, Matsudo 648, Matsudo, Chiba 271-8510, Japan; ecblab2@gmail.com (T.N.); s-hikosaka@faculty.chiba-u.jp (S.H.); goto@faculty.chiba-u.jp (E.G.); 2 Plant Molecular Research Center, Chiba University, Chiba 260-0856, Japan

**Keywords:** antioxidant, bioactive compound, essential oil, plant growth, phytochemical, perillaldehyde, rosmarinic acid, secondary metabolite, UV light, fluorescent lamp

## Abstract

In Japan, red perilla leaves are used in the food and coloring industries, as well as in crude medicine. Perilla leaves contain a high concentration of phytochemicals such as perillaldehyde (PA) and rosmarinic acid (RA). The effects of UV-B radiation intensity (0.05–0.2 W m^−2^, UV-B_BE_: 0.041–0.083 W m^−2^), duration (3 or 6 h), and irradiation method (continuous or intermittent) for artificial nocturnal lighting using UV-B fluorescent lamps were evaluated on growth, flowering, and leaf phytochemical concentration in greenhouse-grown perilla. Under continuous UV-B irradiation at 0.1 W m^−2^ for 3 or 6 h, leaf color changed from red to green and leaf fresh weight decreased, compared with the control treatment. No leaf color change was observed under the 3-h treatment with UV-B radiation at 0.05 W m^−2^, wherein leaf fresh weight was similar to that of the control. Furthermore, RA concentration under continuous UV-B irradiation at 0.05 W m^−2^ for 3 h increased two-fold compared to that under control treatment, while PA concentration was not affected by UV-B irradiation. Thus, our data showed that continuous UV-B irradiation at 0.05 W m^−2^ for 3 h could effectively produce RA-rich perilla leaves without reducing in phenotypic quality or productivity. However, a 6-h intermittent illumination inhibited flowering without altering phytochemical concentration.

## 1. Introduction

As consumers continue to become health-conscious, interest in vegetable phytochemicals has increased. In addition to being recognized as nutrients, phytochemicals are defined as bioactive non-nutrient plant compounds present in fruits, vegetables, grains, and other plant foods [1]. In recent years, research on the effects of phytochemicals exerted on human health has witnessed significant progress and various functionalities have been revealed. Therefore, vegetable growers and companies continue to lay emphasis on vegetable varieties that contain high levels of phytochemicals and to cultivation methods that can be used to promote them.

Red perilla (*Perilla frutescens*) is an annual plant of the family Lamiaceae that has been used in food and coloring industries, and in crude medicine in Japan. Red perilla leaves are rich in phytochemicals such as perillaldehyde (PA) and rosmarinic acid (RA). PA is a characteristic aroma component of red perilla with neuropharmacological [2], vasodilatory [3], and antidepressant-like properties [4], and bactericidal action [5,6]. RA has been shown to exert anti-allergy effects [7,8] and to inhibit amyloid-β (Aβ) aggregation, a protein involved in the development of Alzheimer’s disease [9]. Furthermore, such phytochemicals in red perilla are known to be affected by UV-B radiation. Previous studies conducted in growth chambers have reported that UV-B irradiation influences growth, development, PA, and RA concentration in red perilla plants [10,11]. Additionally, it has been reported that the concentration of certain phytochemicals increases with exposure to UV-B irradiation in other members of Lamiaceae [12,13,14,15,16,17].

Perilla is a short-day plant whose commercial greenhouse production is dependent on the use of lighting with incandescent and fluorescent bulbs to establish photoperiodic control to suppress flowering for continuous leaf production, although recently, the use of LEDs has witnessed an increase. Generally, three to four hours of night-interruption or day-extension lighting is used for greenhouse perilla production. It was reported that flowering causes not only a decrease in leaf production but also a decrease in the concentration of essential oil components such as PA [18,19]. Thus, flowering control is important for maintaining quality. Horibata et al. [20] revealed that continuous blue light irradiation inhibits flowering, which in turn increases PA concentration.

Recently, research has been conducted to control spider mites in greenhouse perilla cultivation using UV-B fluorescent lamps [21,22]. Furthermore, the technique of pest control using UV-B fluorescent lamps has been developed to control the occurrence of powdery mildew in strawberries [23]; moreover, a UV-B fluorescent lamp for pest control has been introduced which is now commercialized as an integrated pest management (IPM) technology for strawberry cultivation in Japan. Once the use of UV-B fluorescent lamps for pest control has been established in perilla production, it is expected to be rapidly adopted for greenhouse perilla production. In addition to the reduction in the use of pesticides and in pest control-related labor, the benefits of application of UV-B irradiation, such as increased leaf phytochemical concentration and effective flowering control, should motivate growers to adopt the system, considering that no additional cost is incurred other than that involved in the replacement of the light source. However, the use of UV-B fluorescent lamps to improve the phytochemical concentration in greenhouse cultivated perilla has not been studied, and its effect on flowering remains unknown. Furthermore, UV-B radiation reportedly exerts negative effects, such as changes in leaf color [21] and reduced growth [10]; therefore, it is necessary to establish irradiation conditions that do not cause a reduction in phenotypic quality and productivity.

In this study, we investigated a UV-B irradiation method that could enhance phytochemical concentration while suppressing flowering and maintaining the quality and productivity of greenhouse-grown red perilla.

## 2. Results

### 2.1. Experiment 1: Exposure to 6 h of Nocturnal UV-B Lighting

#### 2.1.1. Growth, Phenotype, and Flowering

**Figure 1:**
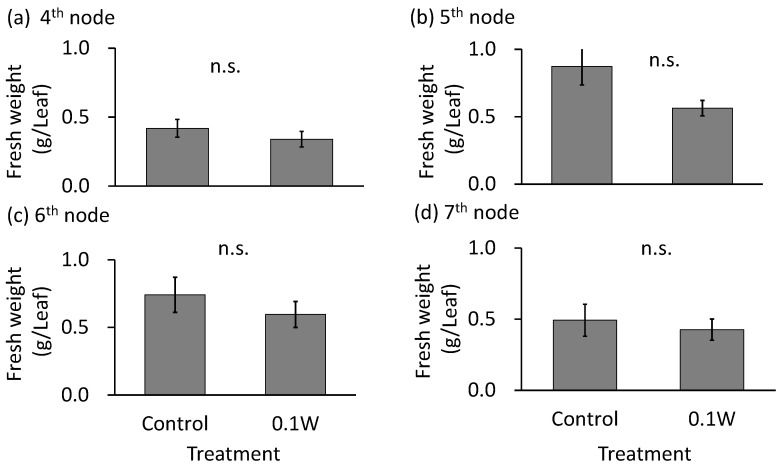
Effect of exposure to 6 h of nocturnal UV-B lighting on leaf fresh weight of red perilla plants (Experiment 1). Perilla plants were irradiated for 6 h from 23:00 to 05:00 h in all UV-B light treatments. UV-B radiation intensity at the top of the plant canopy was set to 0.1 W m^−2^. The integrated UV-B energy at 0.1 W m^−2^ was 2.2 kJ m^−2^ day^−1^. Leaves at the 4th node counted from the bottom of the main stem were harvested 1 week after treatment initiation; thereafter, leaves at the 5th, 6th, and 7th nodes were sampled from the same plants at 2, 3, and 4 weeks after treatment initiation. Vertical bars indicate SE (*n* = 6). n.s. indicates no significant difference as per the *t*-test.

**Figure 2:**
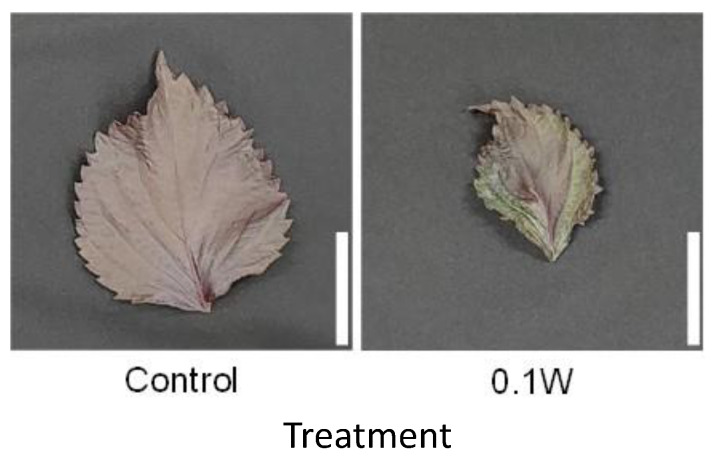
Appearance of a red perilla leaf at the 5th nodes counted from the bottom of the main stem 2 weeks after treatment initiation (Experiment 1). Perilla plants were irradiated for 6 h from 23:00 to 05:00 h in a UV-B light treatment regimen. The UV-B radiation intensity at the top of the plant canopy was set to 0.1 W m^−2^. The integrated UV-B energy at 0.1 W m^−2^ was 2.2 kJ m^−2^ day^−1^. Leaves at the 5th node counted from the bottom of the main stem were harvested 2 weeks after treatment initiation. Leaf blade length under control and under 0.1 W m^−2^ treatment conditions were 11.0 ± 0.7 and 7.9 ± 0.8 cm, respectively (mean ± SE, *n* = 6). Each white bar indicates 5 cm.

The emergence of two leaves from a node on the main stem was observed every week in both treatments. Since the leaves of the 4th node counted from the bottom of the main stem were already one-week old at the time of treatment initiation, they were irradiated with UV-B radiation for only 1 week, while the leaves at the other nodes were irradiated for approximately 2 weeks. There was no significant difference in the fresh weight of leaves at any leaf position (Figure 1). Leaf color changed from purple to green 1 week after initiation of the 0.1-W m^−2^ treatment, and concomitantly, leaf shriveling and curling were observed (Figure 2). Additionally, leaf blade length under conditions of the 0.1-W m^−2^ treatment was found to be decreased compared with that under control conditions. Flowering was observed in all control plants at 4 weeks after treatment initiation, whereas only two plants flowered (33%) under the 0.1-W m^−2^ treatment, at 5 weeks after treatment initiation (Table 1).

#### 2.1.2. Phytochemical Concentration

**Figure 3:**
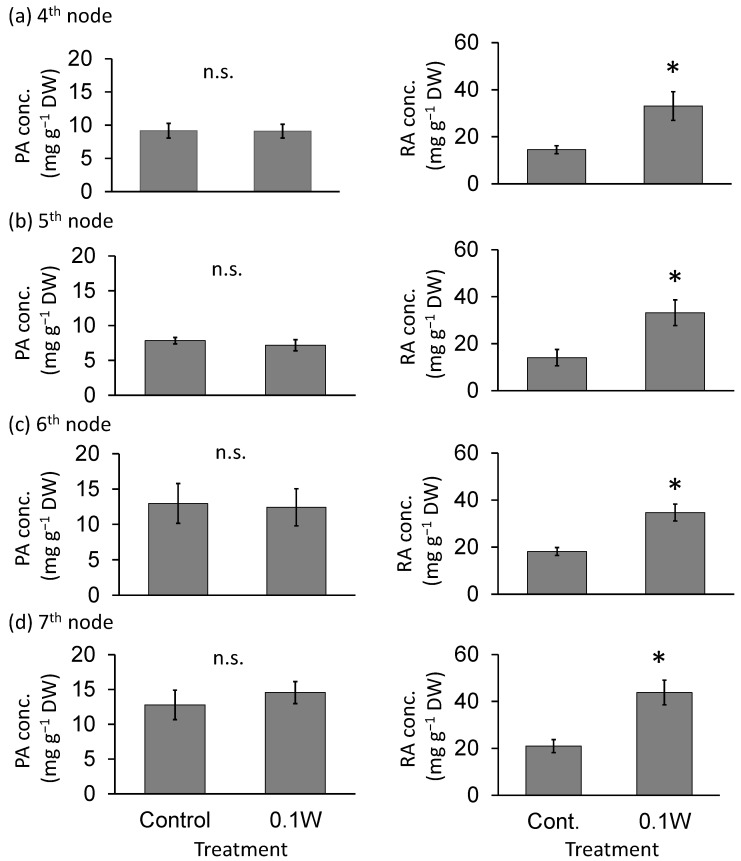
Effect of exposure to 6 h of nocturnal UV-B lighting on phytochemical (perillaldehyde (PA), rosmarinic acid (RA)) concentrations (per dry weight basis) (Experiment 1). Perilla plants were irradiated for 6 h from 23:00 to 05:00 h in all UV-B light treatments. UV-B radiation intensity at the top of the plant canopy was set to 0.1 W m^−2^. The integrated UV-B energy at 0.1 W m^−2^ was 2.2 kJ m^−2^ day^−1^. Leaves at the 4th node counted from the bottom of the main stem were harvested 1 week after treatment initiation; thereafter, leaves at the 5th, 6th, and 7th nodes were sampled from the same plants at 2, 3, and 4 weeks after treatment initiation. Vertical bars indicate SE (*n* = 6). The asterisk symbol indicates significant difference between treatments at *p* < 0.05, and n.s. indicates no significant difference as per the *t*-test.

There was no significant difference in PA concentration, on a per dry weight basis, between treatments (Figure 3). The RA concentration tended to increase with increasing UV-B radiation intensity throughout the treatment period. For example, at the 7th node, 4 weeks after treatment initiation, RA concentration increased 2.1-fold under 0.1 W m^−2^ treatment conditions compared with that under control treatment.

### 2.2. Experiment 2: Exposure to 3 h of Nocturnal UV-B Lighting

#### 2.2.1. Growth, Phenotype, and Flowering

**Figure 4:**
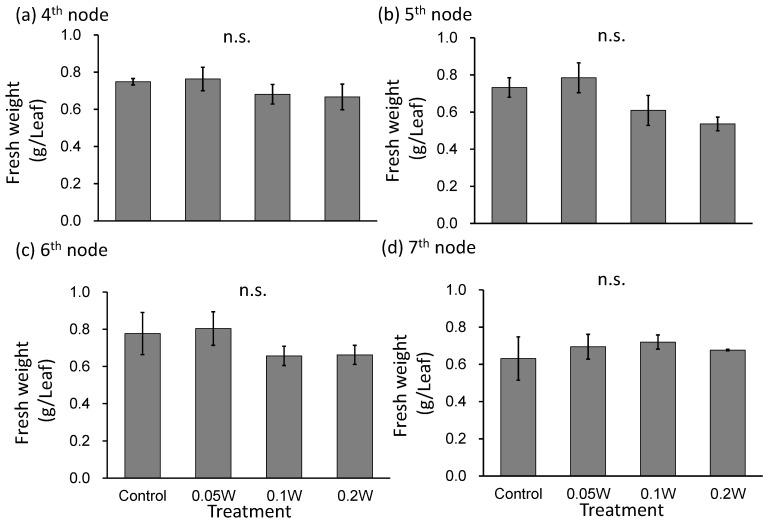
Effect of exposure to 3 h of nocturnal UV-B lighting on leaf fresh weight of red perilla plants (Experiment 2). Perilla plants were irradiated for 3 h from 02:00 to 05:00 in all UV-B light treatments. The UV-B radiation intensity at the plant canopy level was set to 0.05, 0.1, or 0.2 W m^−2^. The integrated UV-B energy at 0.05, 0.1, and 0.2 W m^−2^ was 0.54, 1.0 and 2.2 kJ m^−2^ day^−1^, respectively. Leaves at the 4th node counted from the bottom of the main stem were harvested 1 week after treatment initiation; thereafter, leaves at 5th, 6th, and 7th nodes were sampled from the same plants at 2, 3, and 4 weeks after treatment initiation. Vertical bars indicate SE (*n* = 4). n.s. indicates no significant difference as per the Tukey-Kramer test.

**Figure 5:**
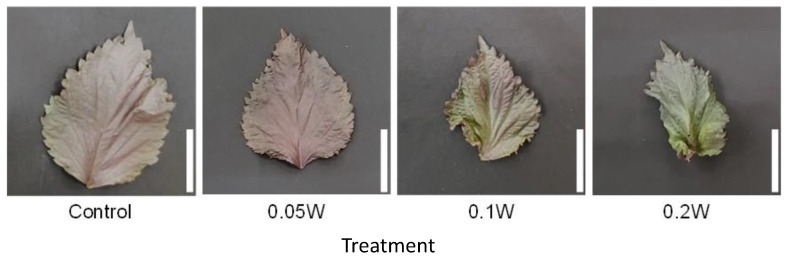
Appearance of a red perilla leaf at the 5th node counted from the bottom of the main stem 2 weeks after treatment initiation (Experiment 2). Perilla plants were irradiated for 3 h from 02:00 to 05:00 h in a UV-B light treatment regimen. The UV-B radiation intensity at the plant canopy level was set to 0.05, 0.1, or 0.2 W m^−2^. The integrated UV-B energy at 0.05, 0.1, and 0.2 W m^−2^ was 0.54, 1.0, and 2.2 kJ m^−2^ day^−1^, respectively. Leaves at the 5th node counted from the bottom of the main stem were harvested 2 weeks after treatment initiation. Leaf blade length under the control, 0.05 W, 0.1 W, and 0.2 W treatment conditions was 11.4 ± 0.8, 11.3 ± 0.4, 9.5 ± 0.4, and 7.1 ± 0.7 cm, respectively (mean ± SE, *n* = 4). Each white bar indicates 5 cm.

Leaf fresh weight tended to be smaller under the 0.1-and 0.2-W m^−2^ treatment conditions than under any other treatment at the 5th and 6th nodes (Figure 4), although there was no growth inhibition due to subjection to UV-B irradiation thereafter. At the 4th node, 1 week after treatment initiation, leaves under the 0.2-W m^−2^ showed a slight change in leaf color, whereas leaves under the other four treatment conditions did not show any change. Two weeks after treatment initiation, change in leaf color was observed under the 0.1-W m^−2^ treatment condition as well as under the 0.2-W m^−2^ treatment condition (Figure 5). As observed for fresh weight, leaf blade length tended to be smaller under the 0.1- and 0.2-W m^−2^ treatment conditions than that observed under any other treatment condition at the 5th node. Flowering was observed in all plants across treatments within 5 weeks after treatment initiation (Table 2). Flowering was delayed under high irradiation treatment conditions.

#### 2.2.2. Phytochemical Concentration

**Figure 6:**
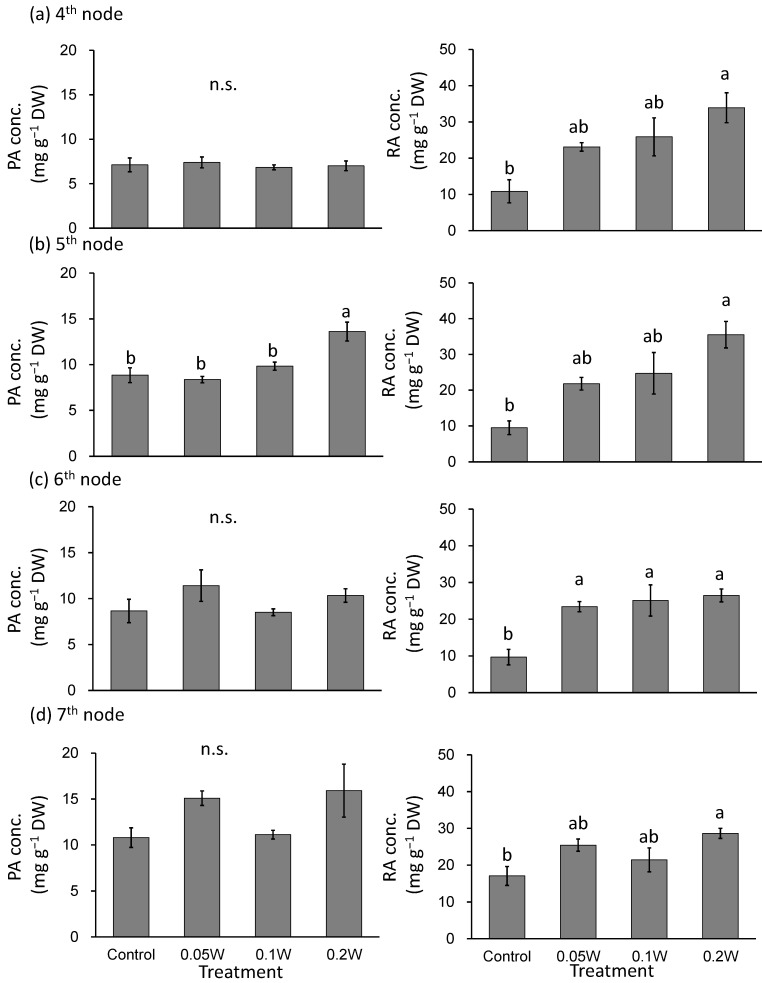
Effect of exposure to 3 h of nocturnal UV-B lighting on phytochemical (perillaldehyde (PA), rosmarinic acid (RA)) concentrations (Experiment 2). Perilla plants were irradiated for 3 h from 02:00 to 05:00 in all UV-B light treatments. The UV-B radiation intensity at the plant canopy level was set to 0.05, 0.1, or 0.2 W m^−2^. The integrated UV-B energy at 0.05, 0.1, and 0.2 W m^−2^ was 0.54, 1.0 and 2.2 kJ m^−2^ day^−1^, respectively. Leaves at the 4th node counted from the bottom of the main stem were harvested 1 week after treatment initiations; thereafter, leaves at the 5th, 6th, and 7th nodes were sampled from the same plants at 2, 3, and 4 weeks after treatment initiation. Vertical bars indicate SE (*n* = 4). Different letters indicate significant difference among treatments at *p* < 0.05, and n.s. indicates no significant difference as per the Tukey-Kramer test.

There was no significant difference in PA concentration at the 4th, 6th and 7th nodes (Figure 6). PA concentration at the 5th node was significantly higher under the 0.2-W m^−2^ treatment condition than that under any other treatment condition. RA concentration tended to be greater under all UV-B treatment conditions than those observed under the control treatment condition at the 4th and 5th nodes, particularly under the higher intensity UV-B treatment conditions. The RA concentration was significantly higher under the 0.05-, 0.1-, and 0.2-W m^−2^ treatment conditions than that observed under control conditions at the 6th node. The RA concentration was significantly greater at the 7th node under the 0.2-W m^−2^ treatment condition than that under the control treatment condition, while there were no significant differences between the other treatments.

### 2.3. Experiment 3: Exposure to Intermittent Nocturnal Lighting for 6 h with UV-B Light

#### 2.3.1. Growth, Phenotype, and Flowering

**Figure 7:**
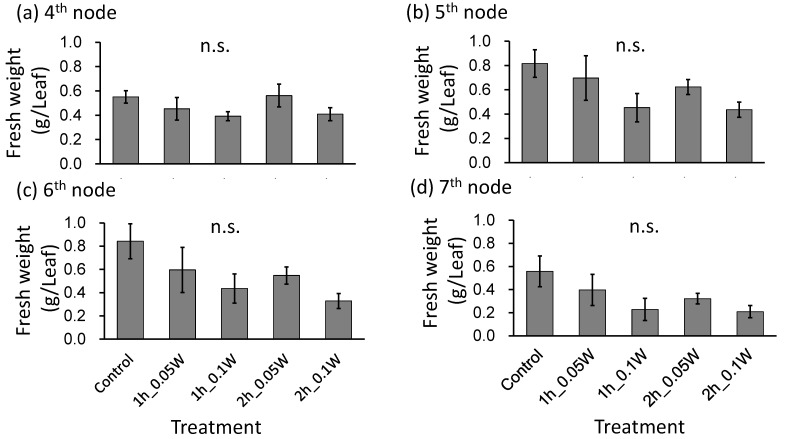
Effect of exposure to intermittent nocturnal lighting for 6 h with UV-B light on leaf fresh weight in red perilla plants (Experiment 3). Perilla plants were irradiated for 1 or 2 h every 1 or 2 h for 6 h, from 18:00 to 06:00. The UV-B radiation intensity at the canopy level was set to 0.05 or 0.1 W m^−2^. The integrated UV-B per night at 0.05 and 0.1 W m^−2^ was 1.1 and 2.2 kJ m^−2^ day^−1^, respectively. Leaves at the 4th node counted from the bottom of the main stem were harvested 1 week after treatment initiation; thereafter, leaves at the 5th, 6th, and 7th nodes were sampled from the same plants at 2, 3, and 4 weeks after treatment initiation. Vertical bars indicate SE (*n* = 5). n.s. indicates no significant difference as per the Tukey-Kramer test.

**Figure 8:**
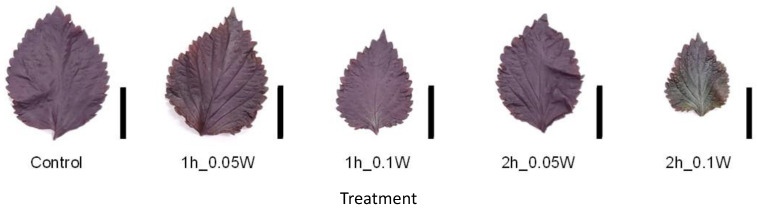
Appearance of a red perilla leaf at the 5th node counted from the bottom of the main stem 2 weeks after treatment initiation (Experiment 3). Perilla plants were irradiated for 1 or 2 h every 1 or 2 h for 6 h, from 18:00 to 6:00. The UV-B radiation intensity at the canopy level was set to 0.05 or 0.1 W m^−2^. Leaves at the 5th node counted from the bottom of the main stem were harvested 2 weeks after treatment initiation. Leaf blade length under treatment conditions of the control, and under conditions of exposure to 0.05 W m^−2^ for 1 h, 0.1 W m^−2^ for 1 h, 0.05 W m^−2^ for 2 h, and 0.1 W m^−2^ for 2 h was 11.3 ± 0.6, 10.2 ± 1.5, 8.4 ± 1.2, 10.5 ± 0.5, and 8.0 ± 0.7 cm, respectively (mean ± SE, *n* = 5). Each black bar indicates 5 cm.

There was no significant difference in fresh weight between treatments (Figure 7). A change in leaf color was only observed under exposure to 0.1 W m^−2^ of intermittent lighting for 2 h (Figure 8). Although leaf shriveling and curling were not observed, leaf blade length under the 0.1-W m^−2^ and 0.1-W m^−2^ treatments for 1 and 2 h, respectively, tended to be smaller than those observed under the other treatment conditions. Flowering was observed in all control plants at 4 weeks after treatment initiation but was suppressed in all intermittent lighting treatments (Table 3).

#### 2.3.2. Phytochemical Concentration

**Figure 9:**
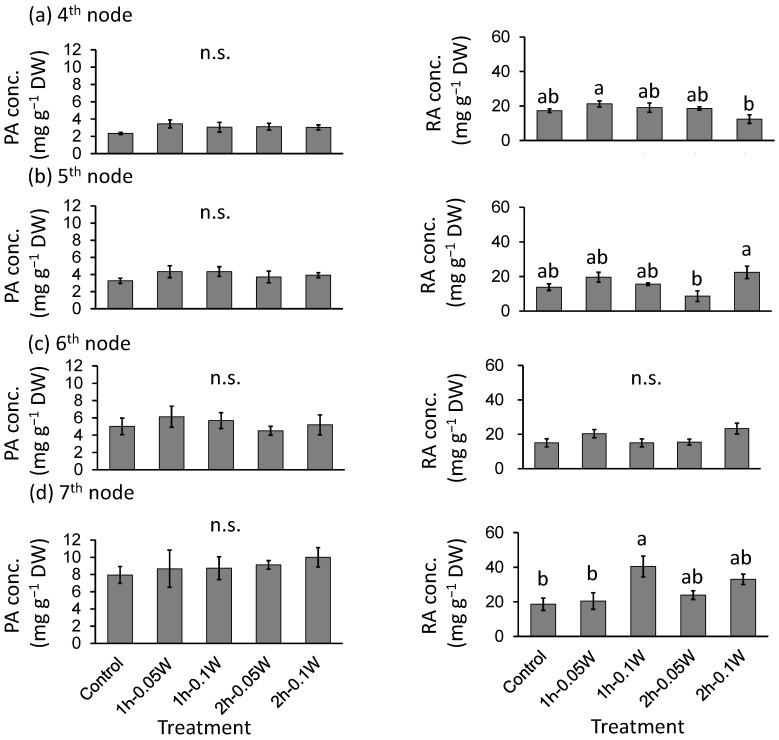
Effect of exposure to intermittent nocturnal lighting for 6 h with UV-B light on phytochemicals (perillaldehyde (PA), rosmarinic acid (RA)) concentrations (Experiment 3). Perilla plants were irradiated for 1 or 2 h every 1 or 2 h for 6 h, from 18:00 to 6:00. The UV-B radiation intensity at the canopy level was set to 0.05 or 0.1 W m^−2^. The integrated UV-B per night at 0.05 and 0.1 W m^−2^ was 1.1 and 2.2 kJ m^−2^ day^−1^, respectively. Leaves at the 4th node counted from the bottom of the main stem were harvested 1 week after treatment initiation; thereafter, leaves at the 5th, 6th, and 7th nodes were sampled from the same plants at 2, 3, and 4 weeks after treatment initiation. Vertical bars indicate SE (*n* = 5). Different letters indicate significant difference among treatments at *p* < 0.05, and n.s. indicates no significant difference as per the Tukey-Kramer test.

There was no significant difference in PA concentration between treatments (Figure 9). The RA concentrations were significantly higher under conditions of exposure to 0.1 W m^−2^ of 1 h intermittent lighting than those observed under any other treatment at the 7th nodes. However, there was no significant difference in RA concentration between treatments at any other leaf position.

### 2.4. Experiment 4: Effect of Visible Light of UV-B Lamps on Flowering

#### Flowering

Flowering was observed in all control plants at 4 weeks after treatment initiation but was suppressed in UV cut treatment (Table 4). A change in leaf color, and in leaf shriveling and curling, was not observed in the UV cut treatment. 

## 3. Discussion

As UV-B light sources are being used as artificial lighting to improve phytochemical concentration and to control flowering, it is desirable to provide illumination to an extent that does not impair growth rate and phenotype quality. Using the same light source as that used in the present study, Ota et al. [21] did not report any effect on plant growth under conditions of exposure to 3 h of illumination at 0.05 W m^−2^ (0.54 kJ m^−2^ day^−1^). In the present study, no growth inhibition via subjection to UV-B irradiation was observed under the same conditions. However, leaf fresh weight decreased upon subjection to UV-B radiation intensities beyond 0.1 W m^−2^. Therefore, for plant growth to remain uninhibited by exposure to 3 h of UV-B irradiation, the light intensity should not exceed 0.05 W m^−2^. In our experiment using intermittent lighting for 6 h daily (experiment 3), plant growth was suppressed under conditions of subjection to irradiation at 0.05 W m^−2^. Therefore, the effect of intermittent lighting on growth inhibition seemed to be more influenced by daily exposure dose rather than by UV-B intensity, and indicated that the exposure dose for normal plant growth should be less than 0.54 kJ m^−2^ day^−1^. Intermittent lighting was expected to confer better protection to plant growth against UV damage during the non-UV irradiation period compared with continuous lighting. However, the results of fresh weight upon subjection to treatment with 2.2 kJ m^−2^ day^−1^ of UV-B, as described in experiments 1–3, showed that intermittent lighting was not effective in preventing damage to leaf growth caused by exposure to UV-B light.

It has been reported that UV-B irradiation decreases the concentration of anthocyanins in red perilla leaves and causes a change in their color to green [10,21,24]. In this study, we observed leaf greening due to UV-B irradiation. However, unlike the findings published by Ota et al. [21], who reported an increase in the intensity of green color in leaves under conditions of exposure to 3 h of 0.05 W m^−2^ irradiation, we observed no change in leaf color under the same conditions. The effect of light at the end of the dark period on perilla flowering is reportedly greater than that at the beginning of the dark period. In this study, lighting was provided from 02:00 to 05:00, before the beginning of the natural light period. In contrast, Ota et al. [21] performed UV-B irradiation from 00:00 to 03:00, which was a different period for irradiation compared with that considered in the present study. It is known that damage to DNA caused by UV-B can be repaired by using light (UV-A or blue light) [25]. In fact, Ota et al. [21] and Ito et al. [24] reported that simultaneous irradiation with visible light and UV-B suppressed leaf greening. In this study, although the irradiations were not simultaneous, their regimens were close to the beginning of the light period, and it might be possible that UV-B damage was photochemically induced for recovery by sunlight. Upon comparing the same 2.2 kJ m^−2^ day^−1^ UV-B exposure treatments between experiments 1, 2, and 3, 0.1 W m^−2^ with 1 h of exposure to intermittent lighting was observed to suppress growth but did not cause greening of the leaves. Therefore, it was suggested that the UV-B irradiation interval, as well as the daily exposure dose, exerted an effect on leaf greening.

Recently, a study using Arabidopsis as a model plant, reported that UV-B light could affect flowering via the photoreceptor UVR8 and expression of related genes [26]. However, our results showed that flowering inhibition was also observed in the treatment which excluded the UV wavelength range. Since the UV-B fluorescent lamps used in this study also emit visible light to alert greenhouse workers to the dangerous UV-B light, it was likely that the inhibition of flowering was not due to UV-B light but rather due to visible light. The following is a discussion presented on flowering inhibition by UV-B fluorescent lamps based on these facts. 

It has been reported that the limit daylength of perilla is 14 h and 20 min, including twilight [27]. In our study, the natural day length in all trials was less than this limit daylength, whereby all control plants showed flowering during the fourth week. Flowering was suppressed via exposure to 6 h of light, compared to subjection to no light treatment, although flowering was observed in a few plants only under conditions of treatment with 0.1 W m^−2^ UV-B radiation. Takimoto and Ikeda [27] reported that flowering was inhibited by a light intensity of 10 lx (approximately 0.17 µmol m^−2^ s^−1^ converted to photosynthetic photon flux density (PPFD) of daytime sunlight) in the latter half of the dark period. The PPFD was 0.17 µmol m^−2^ s^−1^ at 0.1 W m^−2^ (Table 5), although flowering was observed, possibly due to the mutual shading of plants, which was the intensity limit at which certain plants perceived day length. Hamamoto et al. [28] reported that red light surely inhibited flowering in perilla plants compared to that observed with blue light. Therefore, the low proportion of red light (Table 5) was also considered a factor in the lack of inhibition of flowering. On the other hand, the application of intermittent light for 6 h was found to be effective in inhibiting flowering across treatments, and flowering was markedly inhibited even at 0.05 W m^−2^, which was lower than 0.1 W m^−2^. Therefore, intermittent lighting seems to be more effective than continuous lighting for 6 h. In the 3-h light treatment, flowering tended to be delayed in the highly irradiated treatments, although flowering occurred in all treatments by the fifth week. This suggests that, to reliably suppress flowering in case of 3 h of lighting, it may be necessary to introduce light of the wavelength range that is effective in suppressing flowering.

The concentration of PA did not change, whereas that of RA increased under the UV-B irradiation conditions used in this study. Nishimura et al. [10] reported that the concentration of PA did not change after 21 days of UV-B irradiation. In contrast, Goto et al. [11] reported an increase in PA concentration after a short period of UV-B irradiation (3 days), depending on the duration of UV-B irradiation and the leaf growth stage. Furthermore, a correlation between the number of glandular trichomes, in which PA accumulate and PA concentration has been reported [16]. Indeed, UV-B radiation reduces the density of trichomes on the adaxial leaf surface, whereas it increases the number of trichomes on the abaxial leaf surface [29]. While UV-B radiation is necessary for the normal formation of trichomes in basil, there was a substantial increase in the number of broken trichomes 4 days after UV-B irradiation [30]. Hence, it is likely that the effects of short- and long-term UV irradiation on PA accumulation are different. Yoshida et al. [18,19] have reported that flowering causes a decrease in the concentration of essential oil components such as PA. However, a decrease in PA concentration in flowering plants was not observed in the present study, though this decrease might have occurred in leaves of the upper 8th node that were not harvested in the present study. 

Goto et al. [11] reported that when perilla plants were irradiated with UV-B in an artificial climate chamber, the expression levels of RA biosynthesis-related genes, such as PAL and TAT, increased, resulting in an increase in RA concentration. Thus, RA concentration was shown to be increased by UV-B irradiation. In this study, RA concentration was also shown to increase with UV-B irradiation, indicating that UV-B irradiation was effective at improving RA levels in greenhouse-grown perilla. However, the concentration of RA did not increase in Experiment 3 with application of intermittent illumination. It has been reported that reactive oxygen species (ROS) are generated under conditions of exposure to various stress factors, which in turn promote the accumulation of RA. Intermittent illumination suppressed the generation of ROS during the non-irradiation period, which might have resulted in reduced RA accumulation.

These results indicate that exposure to 3 h of illumination at 0.05 W m^−2^ can increase RA concentration without inhibiting plant growth. However, it was not possible to inhibit flowering of perilla under these conditions, and future studies on wavelength, intensity, and irradiation time are necessary. Furthermore, we showed that exposure to 6 h of illumination was necessary to suppress flowering within the range of light intensity tested. Additionally, the effect was stronger with intermittent illumination, which was considered to be more suitable for suppressing UV-B-induced damage exerted on plant growth. We concluded that flowering suppression by the UV-B light source in this study was attributable to the effect of visible light emitted by the UV-B light source.

## 4. Materials and Methods

### 4.1. Plant Material and Growth Conditions

Perilla (*Perilla frutescens* var. crispa ‘Houkouakashiso’ Nakahara Seed Product Co., Ltd., Fukuoka, Japan) seeds were dipped in tap water until rooting and then sown on saturated urethane sponges. Seeds were grown in a controlled-environment room equipped with white fluorescent lamps (FHF32EX-N-H; Panasonic Co., Ltd., Osaka, Japan). The environmental conditions were as follows: 25/20 °C (light/dark period) air temperature, 70% relative humidity, and 16 h/8 h day/night photoperiod. The PPFD from the light source was set to 150 µmol m^−2^ s^−1^ at the canopy level using a quantum sensor (LI-190; Li-COR, Inc., Lincoln, NE, USA). Three weeks after sowing, plants were transplanted into hydroponic containers equipped with air pumps. A commercial nutrient solution (OAT house A treatment; OAT Agrio Co., Ltd., Tokyo, Japan) with EC at 0.9 dS m^−1^ was used. Seedlings were grown under these conditions until the leaves at the 5th node (from bottom to top) were unfolded. The leaves at the 1st–3rd nodes were fully expanded; however, the leaves at the 4th node were not.

### 4.2. Experimental Growth Conditions

**Figure 10:**
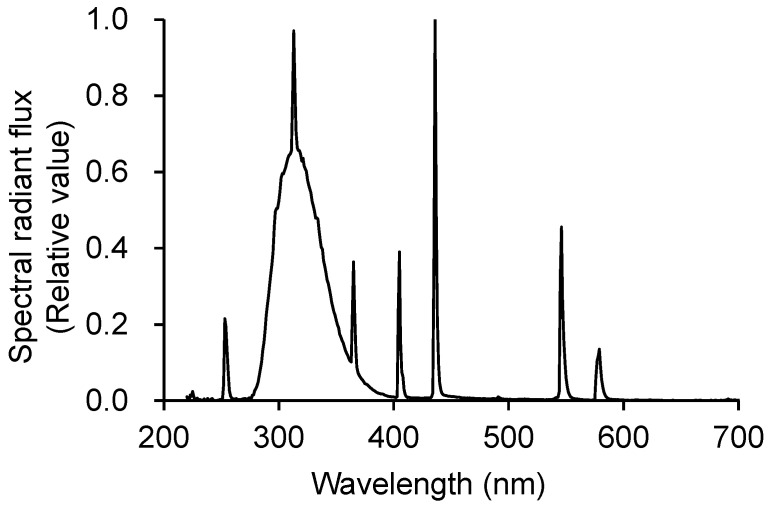
Spectral radiant flux from the UV-B lamp.

Seedlings were transplanted into hydroponic containers equipped with air pumps in an N-S oriented greenhouse. A commercial nutrient solution (OAT house A treatment; OAT Agrio Co., Ltd., Tokyo, Japan) with EC 0.9 at dS m^−1^ was used. Experiments were conducted in a greenhouse located in Matsudo, Chiba, Japan (35.41° N; 139.46° E). We used UV-B fluorescent lamps (SPWFD24UB1PA; Panasonic Lighting Devices Co., Ltd., Takatsuki, Japan). The spectrum of the UV-B lamp was measured using a spectroradiometer (USR-45D; USHIO INC., Yokohama, Japan) (Figure 10). Table 5 lists the spectral characteristics of the lamps. UV-B intensity was measured using a UV radiometer (UV203; Irradian Ltd., Scotland, UK). Biologically effective UV-B radiation (UV-B_BE_) proposed by Caldwell [31] was calculated by using weighting function indicated by Nouchi [32].

#### 4.2.1. Experiment 1: Exposure to 6 h of Nocturnal UV-B Light

Perilla seedlings were grown with or without (control) subjection to UV-B light treatment. Seedlings were irradiated for 6 h from 23:00 to 05:00 h in all UV-B light treatments. Experiment 1 was performed from August 18, 2020, to September 22, 2020 (5 weeks). UV-B intensity at the top of the plant canopy was set to 0.1 W m^−2^ (UV-B_BE_: 0.083 W m^−2^) by adjusting the distance from the light sources to each plant material. The distance was adjusted on a weekly basis. The integrated UV-B energy at 0.1 W m^−2^ was 2.2 kJ m^−2^ day^−1^. 

#### 4.2.2. Experiment 2: Exposure to 3 h of Nocturnal UV-B Light

Seedlings were irradiated for 3 h from 02:00 to 05:00 in all UV-B light treatments. Experiment 2 was performed from September 29, 2020, to November 3, 2020 (5 weeks), at three different levels of UV-B intensity, using UV-B lamps. The UV-B radiation intensity at the plant canopy level was set to 0.05, 0.1, or 0.2 W m^−2^ (UV-B_BE_: 0.041, 0.083, or 0.17 W m^−2^, respectively) by adjusting the distance from the light source to each plant material. The distance was adjusted on a weekly basis. The integrated UV-B energy at 0.05, 0.1 and 0.2 W m^−2^ was 0.54, 1.0 and 2.2 kJ m^−2^ day^−1^, respectively. Based on the results of experiment 1, physiological disorders such as greening of leaves were observed at 0.1 W m^−2^. Therefore, we conducted a 0.05-W m^−2^ treatment in experiment 2. Additionally, we also performed a 0.2-W m^−2^ treatment with the same integrated UV-B energy (2.2 kJ m^−2^ day^−1^) as that at 0.1 W m^−2^ conducted in experiment 1.

#### 4.2.3. Experiment 3: Exposure to Intermittent Lighting for 6 h of Nocturnal UV-B Light

**Figure 11:**
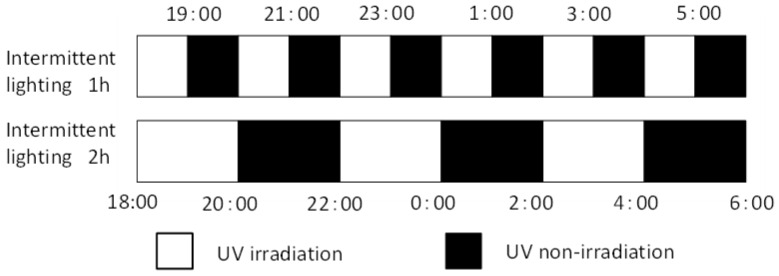
Timing of intermittent UV-B irradiation. White and black bars represent irradiation and non-irradiation by UV-B lamps, respectively. Intermittent UV-B irradiation was conducted for 1 h or 2 h at an interval of 1 or 2 h for 6 h, 18:00 to 6:00. The UV-B radiation intensity at the canopy level was set to 0.05 or 0.1 W m^−2^ by adjusting the distance from the light source to each plant material.

Seedlings were irradiated for 1 or 2 h every 1 or 2 h for 6 h, from 18:00 to 06:00 (Figure 11). Experiment 3 was performed from December 18, 2020, to January 22, 2021 (5 weeks), at two different levels of UV-B intensity using UV-B lamps. The UV-B radiation intensity at the canopy level was set to 0.05 or 0.1 W m^−2^ (UV-B_BE_: 0.041 or 0.083 W m^−2^, respectively) by adjusting the distance from the light source to each plant material. The distance was adjusted on a weekly basis. The integrated UV-B per night at 0.05 or 0.1 W m^−2^ was 1.1 or 2.2 kJ m^−2^ day^−1^, respectively. 

#### 4.2.4. Experiment 4: Effect of Visible Light of UV-B Lamps on Flowering

**Figure 12:**
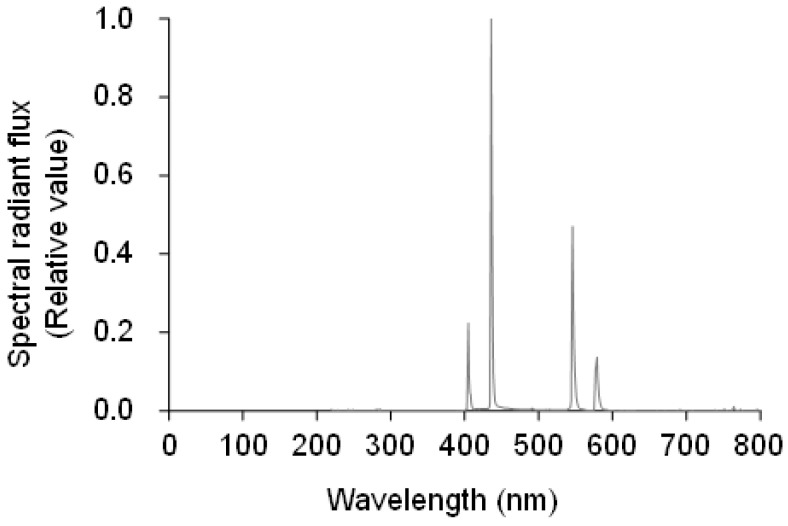
Spectral radiant flux from the UV-B lamp covered with the UV cut film.

Experiment 4 was performed at the same time as experiment 3. The position of a UV-B lamp was adjusted to set the UV-B radiation intensity at the canopy to 0.1 W m^−2^. To evaluate the effect of the visible light of UV-B lamps on flowering, we covered the lamp with a UV cut film (Cut ace, Mitsubishi chemical agri dream Co., Ltd., Tokyo, Japan). The spectrum was measured using a spectroradiometer (USR-45D; USHIO INC., Yokohama, Japan) (Figure 12). Seedlings were irradiated for 6 h from 18:00 to 06:00. 

### 4.3. Leaf Harvesting

The phyllotaxis of perilla plants is decussate. Thus, each node has two leaves at the same growth stage. Leaves at the 4th node counted from the bottom of the main stem were harvested 1 week after treatment initiation; thereafter, leaves at the 5th, 6th, and 7th nodes were sampled from the same plants at 2, 3, and 4 weeks after treatment initiation. One leaf at each node was used to measure the PA and RA content, and the other leaf was used to calculate the dry matter ratio by dividing leaf dry weight by leaf fresh weight. The fresh weights of both leaves were measured immediately after harvesting. Thereafter, one leaf for the measurement of the PA and RA contents was placed in a polyethylene bag, snap frozen in liquid nitrogen, and then stored in a freezer at −30 °C. Thereafter, the leaf used for calculation of the dry matter ratio was oven-dried at 80 °C for 72 h, and then the dry weight was measured. 

### 4.4. Measurement of PA and RA Contents

PA and RA contents were measured according to the method reported by Ogawa et al. [33]. Briefly, 1.0 mL of methanol in a 2.0 mL microtube was added to 0.05 g of leaf tissue ground in liquid nitrogen. Then, the solvents were sonicated in an ultrasonic cleaning bath (ASU-2; AS ONE Corp., Osaka, Japan) for 30 min (10–15 °C) and centrifuged for 10 min (4 °C, 20,380× *g*), and the supernatants were collected. Methanol (0.5 mL) was added to the residue, and sonication and centrifugation were performed as per methods described in the first step. The supernatants obtained from the execution of two steps were combined and centrifuged for 10 min (4 °C, 20,380× *g*), and then filtered using disposable syringe filter units (13HP020AN; Advantec, Tokyo, Japan). 

The PA and RA contents were measured using a UHPLC (Nexera, Shimadzu Corporation, Kyoto, Japan) equipped with a UPLC BEH C18 column (φ1.7 μm, 2.1 mm × 50 mm, Waters Corporation, Milford, MA, USA). The temperature of the column oven was maintained at 50 °C. The detection wavelengths of PA and RA were set at 230 nm and 326 nm, respectively.

### 4.5. Statistical Analysis

Data were statistically evaluated by performing one-way analysis of variance (ANOVA) using the SPSS program for Windows (Version 24.0; SPSS Inc., Chicago, IL, USA). To investigate significant differences among treatments, the mean value of measured data was compared using the *t*-test or the Tukey-Kramer test at *p* < 0.05.

## Figures and Tables

**Table 1:** Weekly percentage flowering of red perilla (Experiment 1, *n* = 6). The weekly percentage flowering was determined by dividing the number of plants that formed flower buds by the number of plants tested. Perilla plants were irradiated for 6 h from 23:00 to 05:00 h in a UV-B light treatment regimen. UV-B radiation intensity at the top of the plant canopy was set to 0.1 W m^−2^.

Treatment	Weeks				
	1	2	3	4	5
Control	0	0	0	100	100
0.1 W	0	0	0	0	33

**Table 2:** Weekly percentage flowering of red perilla (Experiment 2, *n* = 4). The weekly percentage flowering was determined by dividing the number of plants that formed flower buds by the number of plants tested. Perilla plants were irradiated for 3 h from 02:00 to 05:00 in all UV-B light treatments. The UV-B radiation intensity at the plant canopy level was set to 0.05, 0.1, or 0.2 W m^−2^.

Treatment	Weeks				
	1	2	3	4	5
Control	0	0	0	100	100
0.05 W	0	0	0	100	100
0.1 W	0	0	0	50	100
0.2 W	0	0	0	0	100

**Table 3:** Weekly percentage flowering of red perilla (Experiment 3, *n* = 5). The weekly percentage flowering was determined by dividing the number of plants that formed flower buds by the number of plants tested. Perilla plants were irradiated for 1 or 2 h every 1 or 2 h for 6 h, from 18:00 to 06:00. The UV-B radiation intensity at the canopy level was set to 0.05 or 0.1 W m^−2^.

Treatment	Weeks				
	1	2	3	4	5
Control	0	0	0	100	100
1 h-0.05 W	0	0	0	0	0
1 h-0.1 W	0	0	0	0	0
2 h-0.05 W	0	0	0	0	0
2 h-0.1 W	0	0	0	0	0

**Table 4:** Weekly flowering ratio of red perilla (Experiment 4, *n* = 5). The UV cut treatment was conducted by covering the UV-B lamp with a UV cut film to evaluate the effect of the visible light of UV-B lamps on flowering. Before covering the lamp with the film, the UV-B radiation intensity at the canopy level was set to 0.1 W m^−2^. The weekly percentage flowering was determined by dividing the number of plants that formed flower buds by the number of plants tested. Perilla plants were irradiated for 6 h from 18:00 to 06:00.

Treatment	Weeks				
	1	2	3	4	5
Control	0	0	0	100	100
UV cut	0	0	0	0	0

**Table 5:** Spectral characteristic of the UV-B lamp. (UV-B intensity set at 0.1 W m^−2^).

**Wavelength (nm)**	**Intensity (W m^−2^)**
220–280 (UV-C)	0.01
280–315 (UV-B)	0.10
315–380 (UV-A)	0.13
400–700 (PAR)	0.04
**Wavelength (nm)**	**PPFD (µmol m^−2^ s^−1^)**
400–500 (B)	0.093
500–600 (G)	0.073
600–700 (R)	0.004
Total PPFD *	0.169

* Photosynthetic photon flux density.

## Data Availability

Data is contained within the article.
